# Correction to “Dissecting Causal Relationships Between Antihypertensive Drug, Gut Microbiota, and Type 2 Diabetes Mellitus and Its Complications: A Mendelian Randomization Study”

**DOI:** 10.1111/jch.70156

**Published:** 2025-10-17

H. Zheng, S. Wu, W. Wang, W. Qiu, Y. Feng. Dissecting Causal Relationships Between Antihypertensive Drug, Gut Microbiota, and Type 2 Diabetes Mellitus and Its Complications: A Mendelian Randomization Study. *Journal of Clinical Hypertension* 27 no. 1 (2025): e14968. https://doi.org/10.1111/jch.14968

The original version of this article was revised: changes include adjusting the **author affiliation order** and **funding information** to reflect updated institutional details.

The primary **affiliation** is now: “Guangdong Provincial Key Laboratory of Coronary Heart Disease Prevention, Guangdong Cardiovascular Institute, Guangdong Provincial People's Hospital (Guangdong Academy of Medical Sciences), Southern Medical University, Guangzhou, 510080, China.” The secondary affiliation is “School of Medicine, South China University of Technology, Guangzhou, China.”

**Funding** statement was updated to: This work was supported by the Noncommunicable Chronic Diseases‐National Science and Technology Major Project of China (no. 2023ZD0508906 and no. 2024ZD0526803), The Climbing Plan of Guangdong Provincial People's Hospital (DFJH2020022), Guangdong Special Funds for Science and Technology Innovation Strategy (Stability support for scientific research institutions affiliated to Guangdong Province‐GDCI 2024), and The Key Area R&D Program of Guangdong Province (no. 2019B020227005).

We apologize for this error.

